# Supplementary material for: Atomically dispersed nickel as coke-resistant active sites for methane dry reforming
Source: Nat Commun. 2019 Nov 15;10:5181. doi: 10.1038/s41467-019-12843-w (PMC6858327; doi:10.1038/s41467-019-12843-w)
Supplement: Supplementary file 2 — Supplementary material [file 41467_2019_12843_MOESM2_ESM.pdf]

Atomically dispersed nickel as coke-resistant active sites for methane dry reforming

Akri et al.

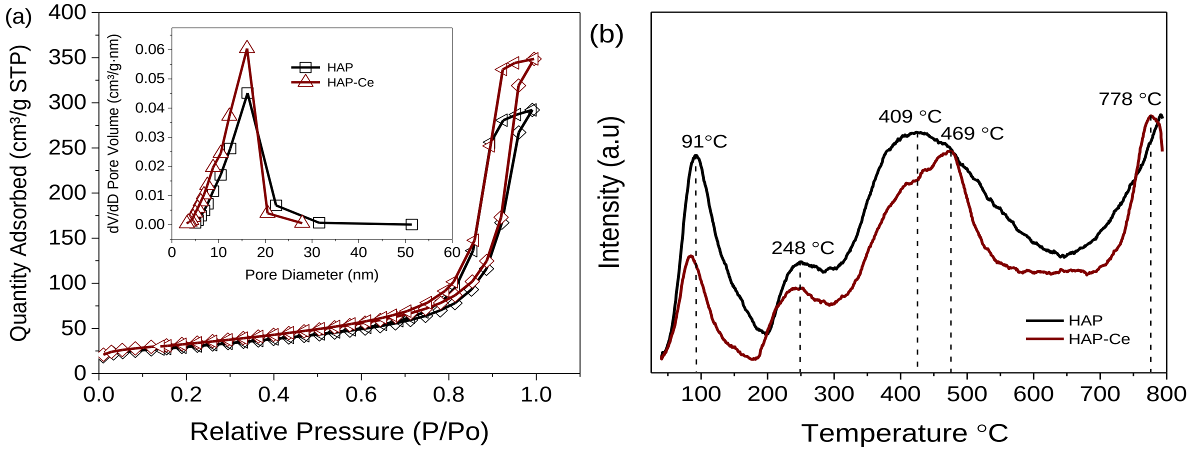

Supplementary Figure 1. Textural and basicity measurements of HAP and HAP-Ce supports. **a** N_2 Adsorption-desorption isotherms and pore size distributions of HAP and HAP-Ce. **b** Basicity measurements by CO_2 adsorption and CO_2-TPD experiments.

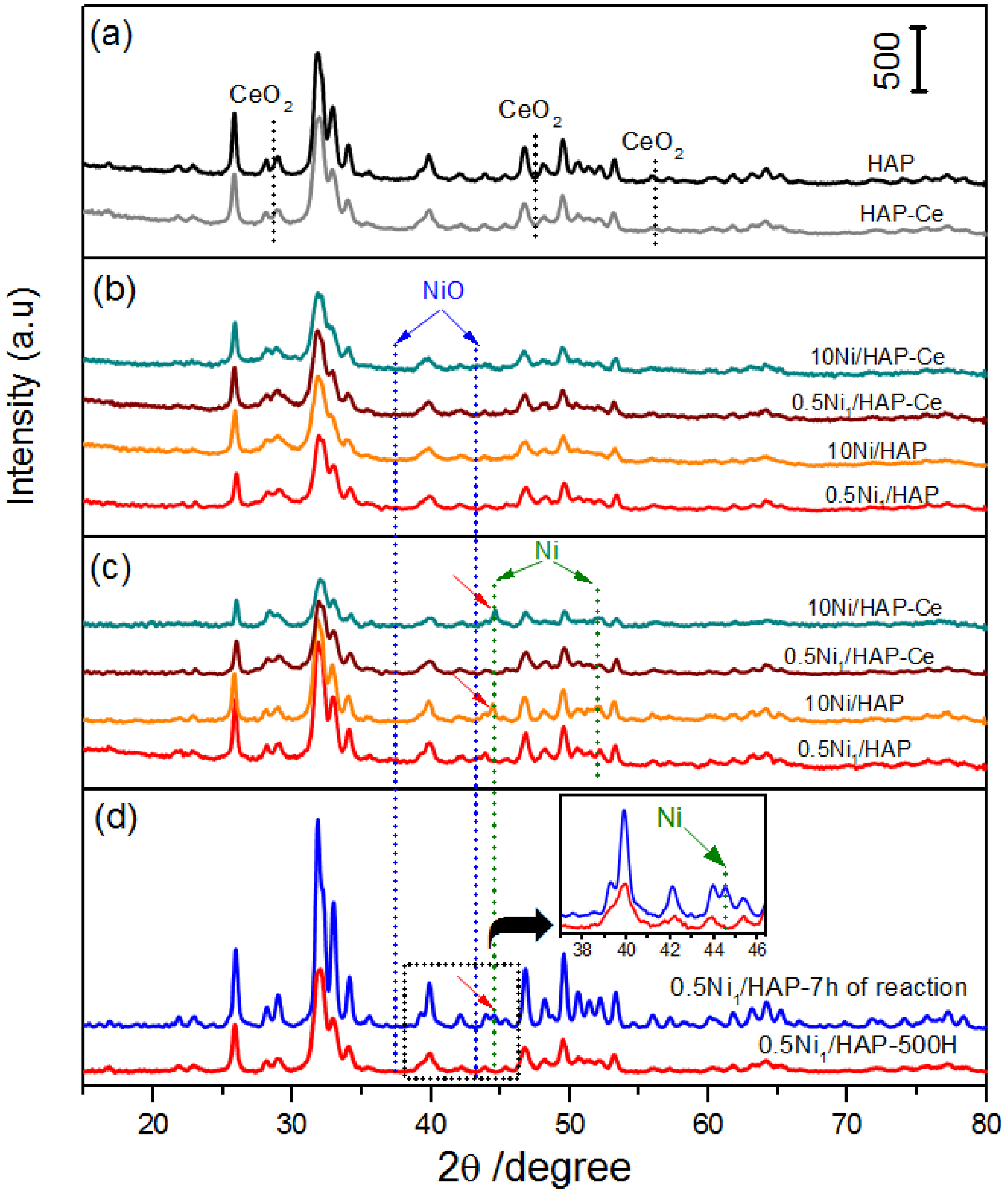

Supplementary Figure 2. Structural properties of supports and catalysts. a XRD patterns of HAP and HAP-Ce supports, **b** HAP and HAP-Ce supported Ni catalyst without reduction, and **c** HAP and HAP-Ce supported Ni catalysts reduced at 500 °C, **d** $0.5Ni_1/HAP$ reduced at 500 °C and after 7 hours of reaction at 750 °C.

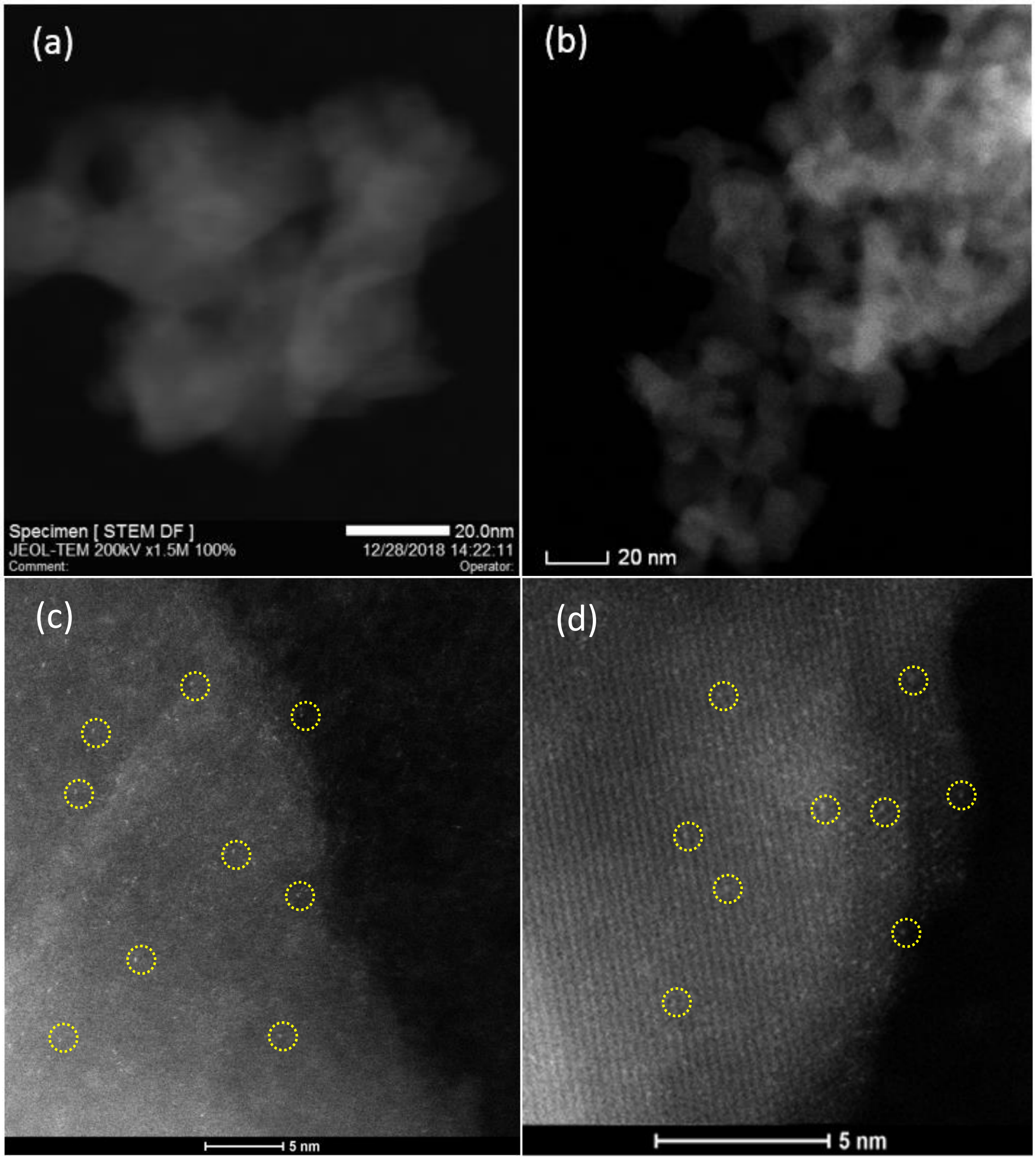

Supplementary Figure 3. Representative STEM images of samples. **a** $0.5Ni_1$/HAP and **b** $0.5Ni_1$/HAP-Ce without reduction, and AC HAADF-STEM images of **c** $0.5Ni_1$/HAP and **d** $0.5Ni_1$/HAP-Ce without reduction. Yellow circles highlight isolated nickel atoms.

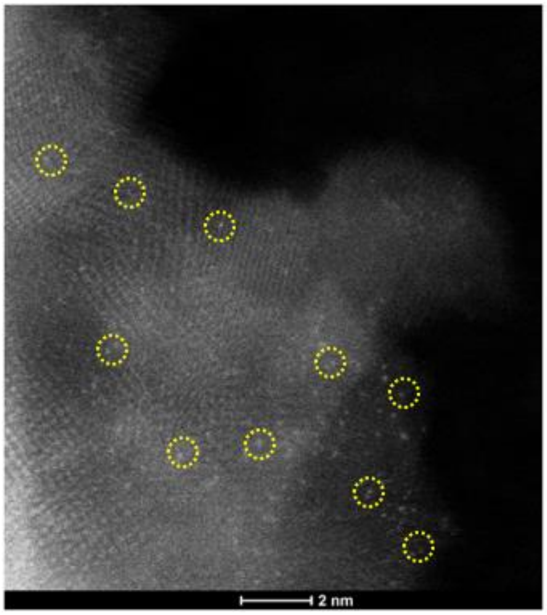

Supplementary Figure 4. AC HAADF-STEM image of $0.5Ni_1$/HAP-Ce reduced at 500 °C. Yellow circles highlight isolated nickel atoms.

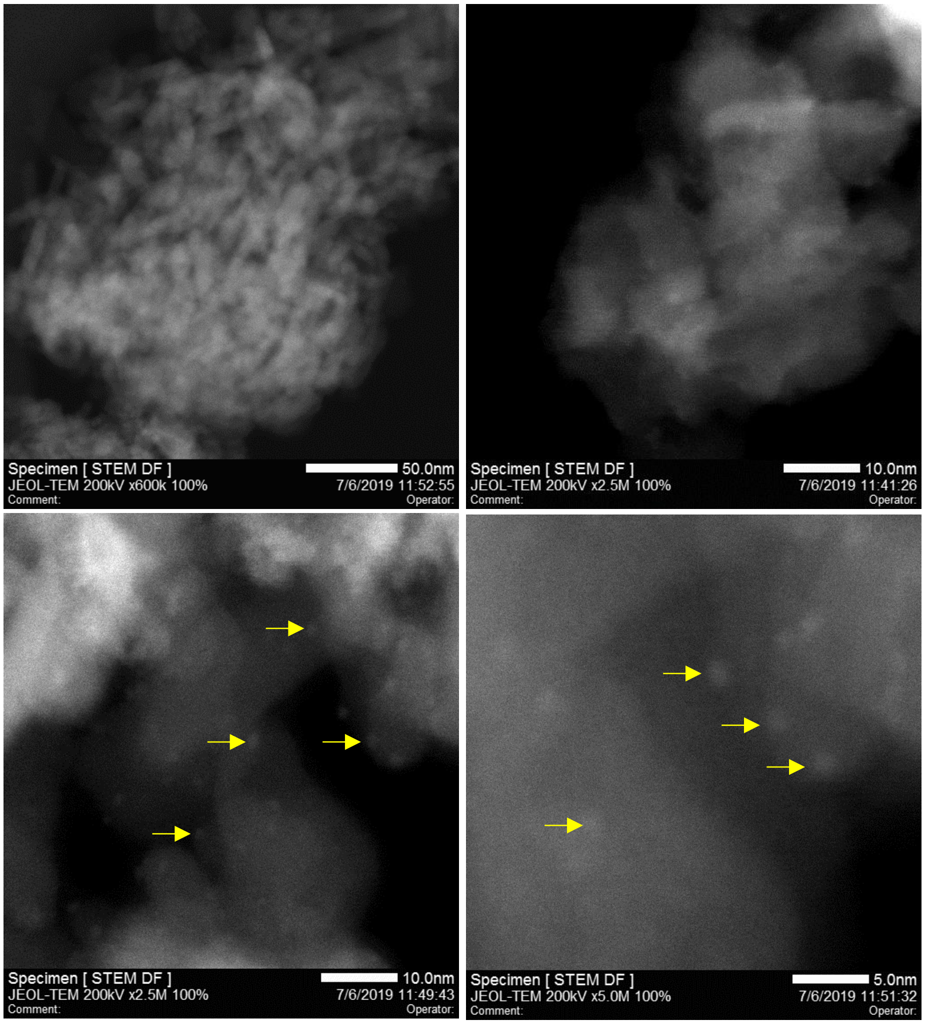

Supplementary Figure 5. Representative STEM images of $1Ni_1$/HAP-Ce after reduction at 500 °C. Yellow arrows highlight nickel nanoclusters.

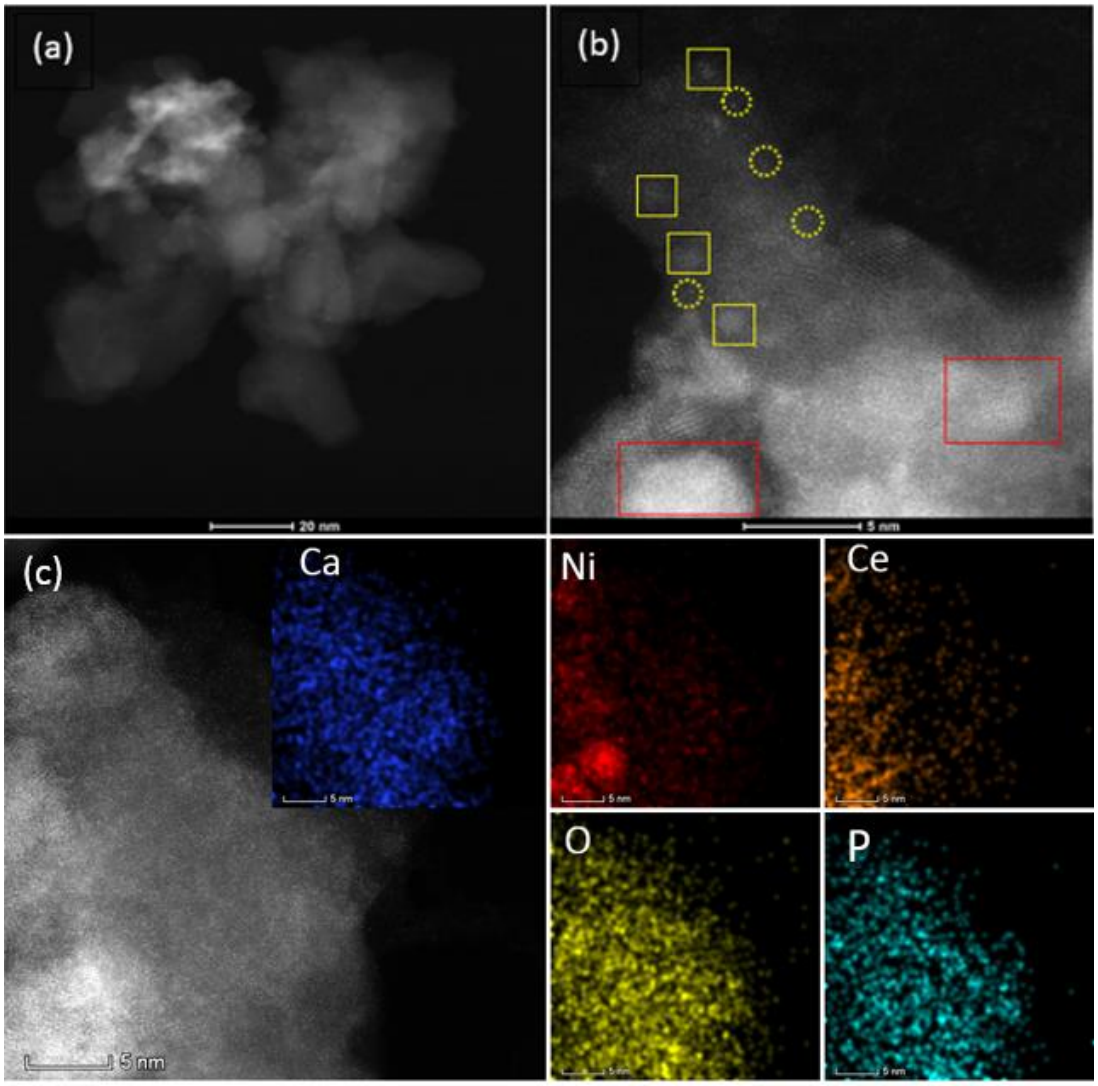

Supplementary Figure 6. Atomic resolution HAADF-STEM images and EDX elemental mapping. a-b Aberration-corrected STEM images of $2Ni_1$/HAP-Ce after reduction at 500 °C. Yellow circles and squares represent nickel single atoms and nanoclusters respectively. Red squares represent nanoparticles. **c** Dark-field scanning TEM image and EDX elemental mapping of $2Ni_1$/HAP-Ce-500 °C-H_2.

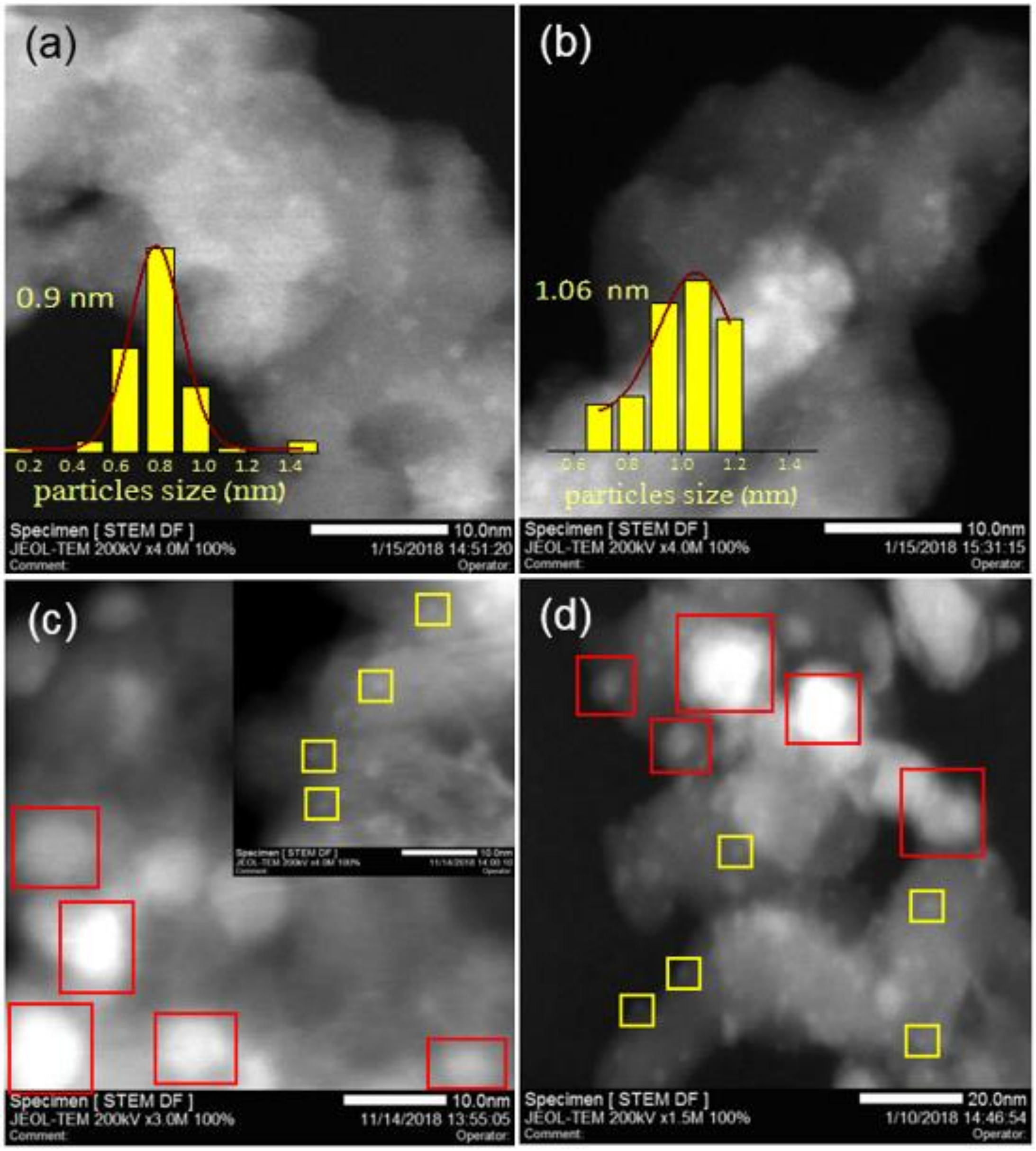

Supplementary Figure 7. STEM images and size distribution of Ni/HAP and Ni/HAP-Ce. **a** 10Ni/HAP and **b** 10Ni/HAP-Ce without reduction, and **c** 10Ni/HAP-Ce and **d** 10Ni/HAP after reduction at 500 °C, respectively. Yellow and red squares highlight nickel nanoclusters and nanoparticle, respectively.

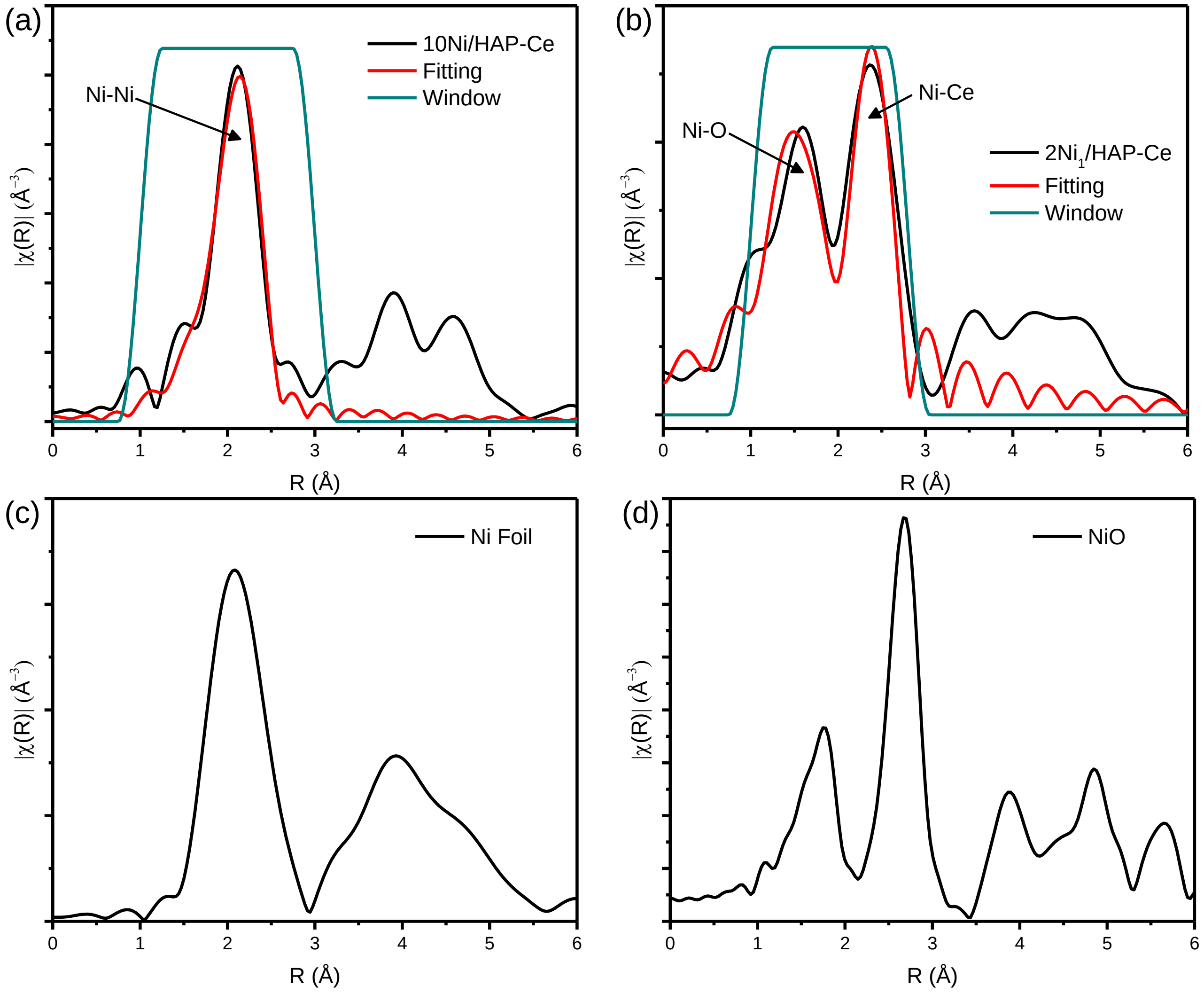

Supplementary Figure 8. XAS results of Ni/HAP-Ce samples. a Ni K-edge EXAFS k^2-weighted Fourier transform magnitudes of 500 °C reduced 10Ni/HAP-Ce, **b** 500 °C reduced 2Ni$_1$/HAP-Ce and **c** Ni foil , and **d** NiO. Experimental data are shown in black and fits in red. All r-space spectra are shown without phase correction.

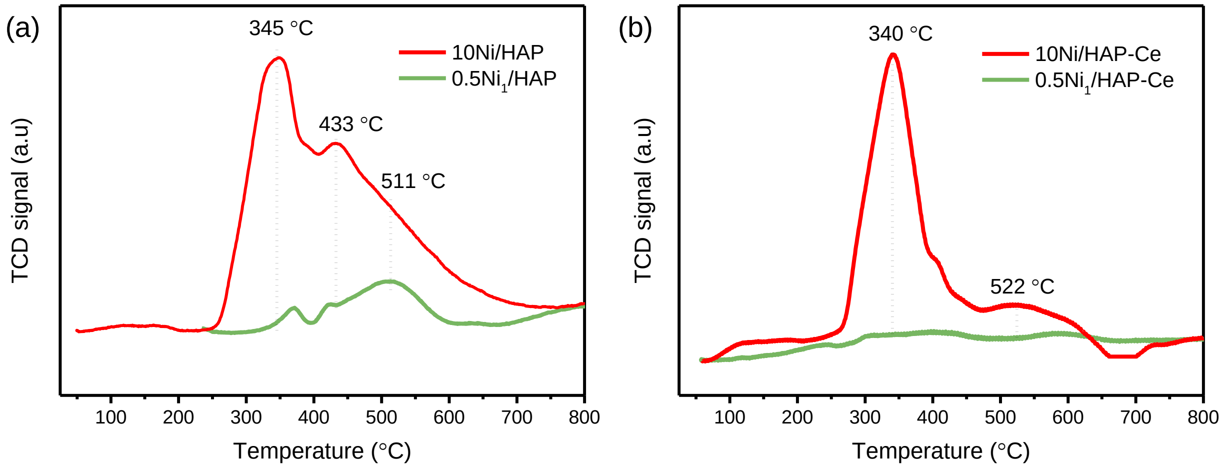

Supplementary Figure 9. H_2-TPR profiles of Ni/HAP and Ni/HAP-Ce. a HAP, and **b** HAP-Ce supported 0.5 wt% and 10 wt% Ni catalysts.

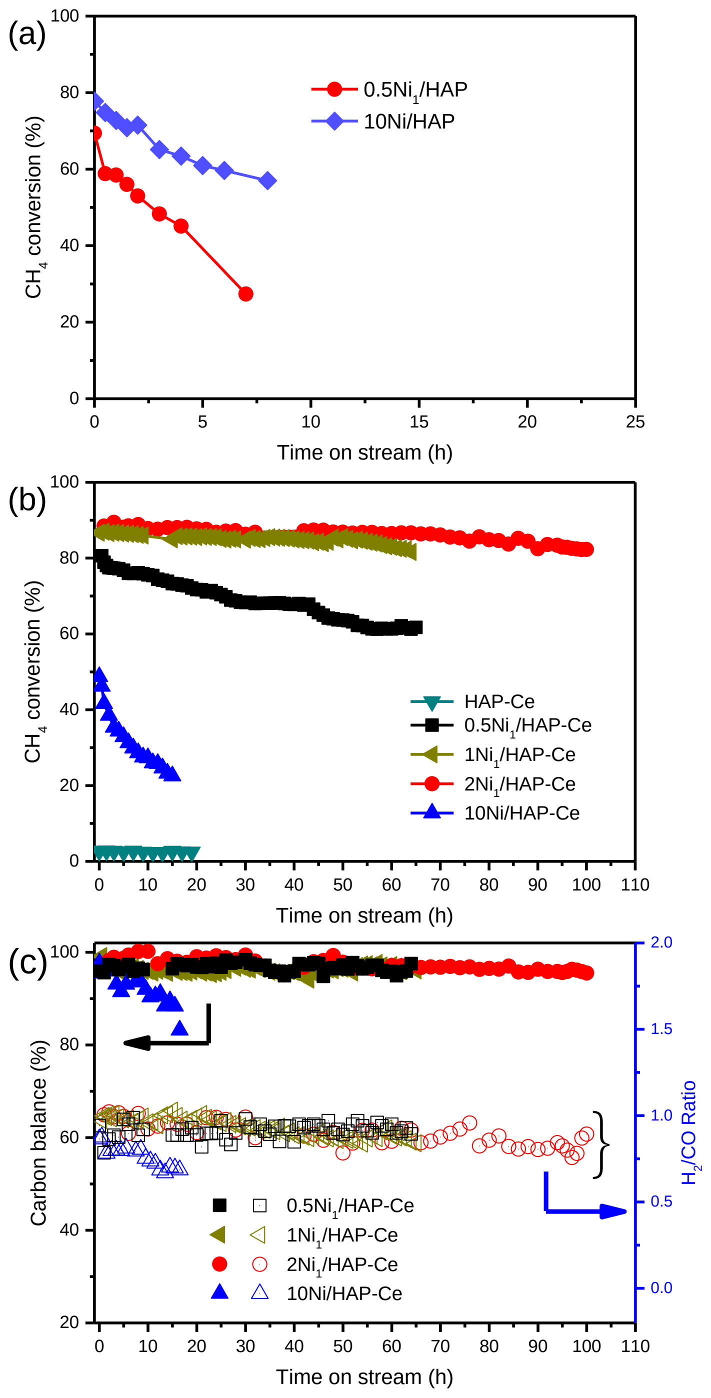

Supplementary Figure 10. Activity and stability of Ni/HAP and Ni/HAP-Ce samples. a CH_4 conversion during dry reforming of methane over HAP supported Ni catalysts and **b** HAP-Ce supported Ni catalysts. Conditions: T=750 °C, $CH_4/CO_2/He$ = 10/10/30, total flow = 50 mL min^{-1}. GHSV = 60,000 mL h^{-1} g^{-1}. **c** Carbon balance and H_2/CO ratio over 0.5Ni$_1$/HAP-Ce, 2Ni$_1$/HAP-Ce, and 10Ni/HAP-Ce supported Ni catalysts during DRM. The carbon balance was calculated from: *Carbon Balance (%)* = $\frac{[CH_4]^{outlet}+[CO_2]^{outlet}+[CO]^{outlet}}{[CH_4]^{Inlet}+[CO_2]^{Inlet}}$.

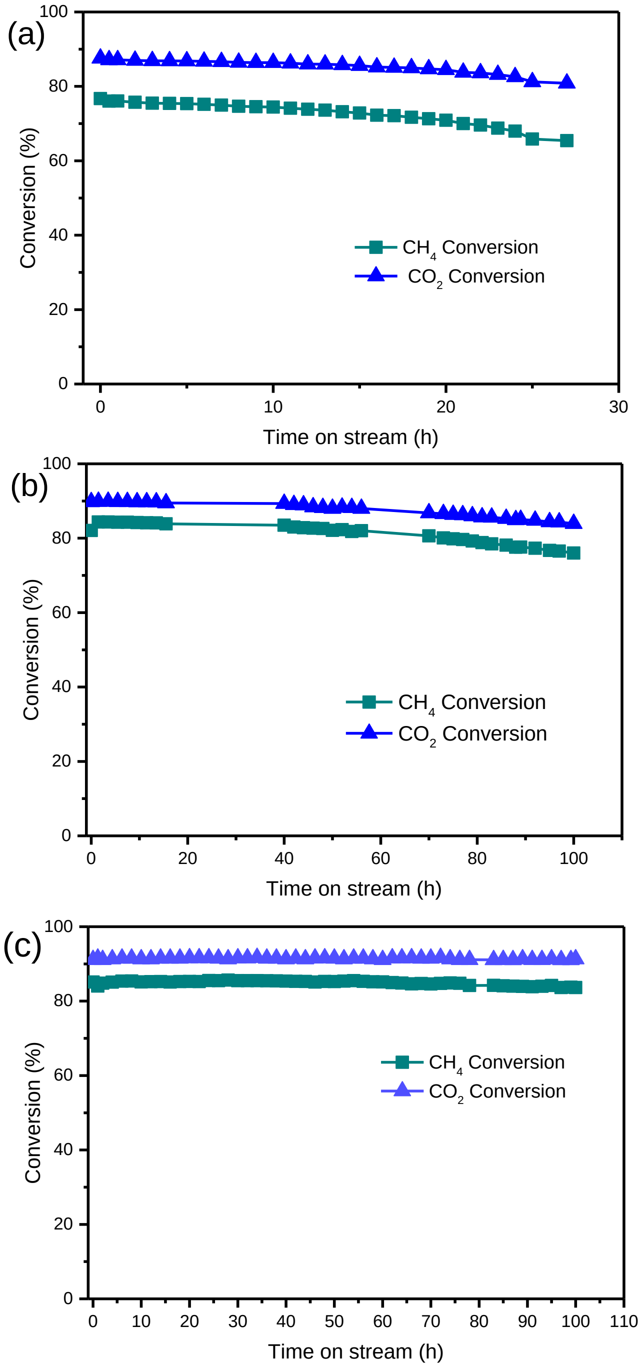

Supplementary Figure 11. Reduction and reaction temperature effect on the performance of Ni/HAP-Ce catalysts. a CO_2 and CH_4 conversion during DRM over $0.5Ni_1$/HAP-Ce and **b** $2Ni_1$/HAP-Ce catalysts reduced at 750 °C. Conditions: T=750 °C, CH_4/CO_2/He = 10/10/30, total flow = 50 mL min–1 (GHSV = 60,000 mL h-1 gcat-1), **c** CO_2 and CH_4 conversion during DRM over $2Ni_1$/HAP-Ce, Conditions: T=600 °C, CH_4/CO_2/He = 1/1/48, total flow = 50 mL min–1 (GHSV = 60,000 mL h^{-1} $gcat^{-1}$).

$$CH_4 \xrightarrow{\text{Step 1}} CH_3\cdot + H\cdot \xrightarrow{\text{Step 2}} CH_2\cdot + H_2 \xrightarrow{\text{Step 3}} CH\cdot + H\cdot + H_2 \xrightarrow{\text{Step 4}} C + 2H_2$$

Supplementary Figure 12. Stepwise, direct C-H bond cleavage during CH_4 decomposition.

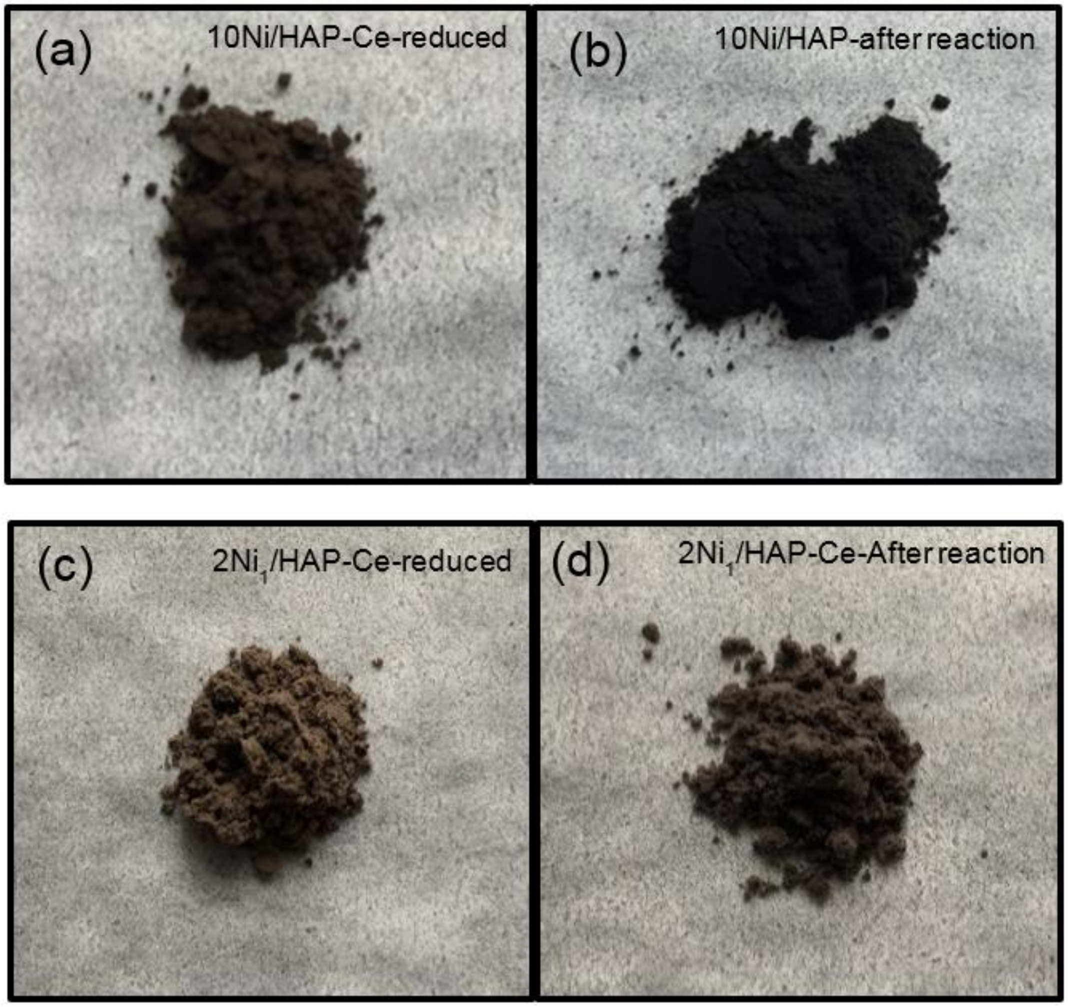

Supplementary Figure 13. Photographs of Ni/HAP-Ce samples. a freshly reduced $10Ni_1$/HAP-Ce and **c** 2Ni/HAP-Ce, and (**b** and **d**) after 1 h of reaction at 750 °C in the presence of CH_4.

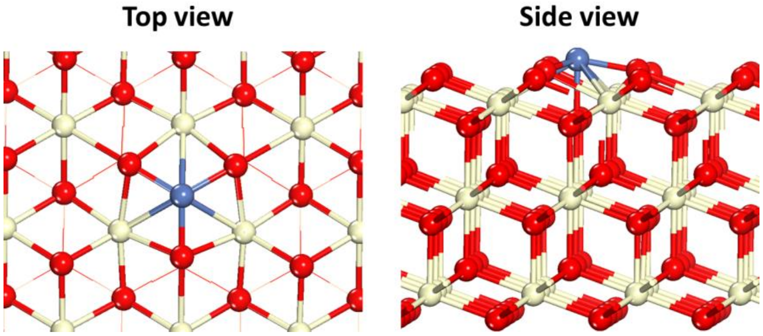

Supplementary Figure 14. Optimized structures of Ni_1/CeO_2 surface. (Ce: yellow, Ni: blue, O: red).

Supplementary Table 1. Nominal and actual loading wt% of Ni/HAP and Ni/HAP-Ce samples.

Catalysts	Nominal loading wt%	Actual loading wt%
**0.5Ni_1/HAP**	0.5	0.64
**10Ni/HAP**	10	10.85
**0.5Ni_1/HAP-Ce**	0.5	0.49
**2Ni_1/HAP-Ce**	2	2.58
**10Ni/HAP-Ce**	10	8.46

Supplementary Table 2. BET surface area and pore volume of HAP and Ce-substituted HAP.

Samples	BET (m^2 g^{-1})	Vp (cm^3 g^{-1})	Pore diameter (nm)
**HAP**	104.8	0.45	14.7
**HAP-Ce**	124.5	0.52	13.9

Supplementary Table 3. Ni K-edge XAFS fitted parameters for $2Ni_1$/HAP-Ce, 10Ni/HAP-Ce, and references.

Samples	Shell	CN	R (Å)	σ^2x10^2 (Å^2)	ΔE_0 (eV)	R-factor (%)
**Ni Foil**	Ni-Ni	12.0	2.48	0.6	4.5	0.001
**NiO**	Ni-O	6.0	2.08	0.4	12.8	0.02
	Ni-Ni	12.0	2.95	0.6	9.1	
**10Ni/HAP-Ce**	Ni-O	2.8	2.09	0.4	0.7	0.3
	Ni-Ni	6.8	2.50	0.6	-5	
**$2Ni_1$/HAP-Ce**	Ni-O	5.7	2.05	0.3	-2.9	1.9
	Ni-Ni	0.7	2.52	0.5	-8.6	
	Ni-Ce	3.9	2.60	0.5	-8.6	

CN = coordination number; R = interatomic scattering distance; σ = Debye-Waller disorder factor; ΔE_0 = absorption edge offset.

Supplementary Table 4. H_2 consumption and reducibility of Ni species in various catalysts.

Catalysts	Nominal loading wt%	Actual loading wt%	H_2 consumption for NiO reduction (mL STP g^{-1}) ± (2%)		NiO Reducibility (%)
			Theoretical amount [a]	Determined [b]	
**$0.5Ni_1$/HAP**	0.5	0.64	2.4	1.4	58
**10Ni/HAP**	10	10.85	41.4	39.8	96
**$0.5Ni_1$/HAP-Ce**	0.5	0.49	1.9	N.D. [c]	---
**10Ni/HAP-Ce**	10	8.46	32.3	36.2	100

[a] Estimated using the actual Ni loadings according to the following reaction: $NiO + H_2 = Ni + H_2O$;
[b] Determined by integrating the area of H_2-TPR peaks;
[c] not detected.
